# Effects of organic mulching on moisture and temperature of soil in greenhouse production of tomato under unheated greenhouse cultivation in the cold zone of China

**DOI:** 10.1002/fsn3.3460

**Published:** 2023-06-01

**Authors:** Pengfei Zhang, Zhaorui Zhang, Menglin Xiao, Jinlong Chao, Yanyan Dai, Geng Liu, Masateru Senge

**Affiliations:** ^1^ School of Geography Science Taiyuan Normal University Jinzhong P. R. China; ^2^ Institute Research of Carb Neutralization Taiyuan Normal University Taiyuan P. R. China; ^3^ Union‐Infrastructure Maintenance Laboratory Gifu University Gifu Japan

**Keywords:** fruit quality, organic mulch hydrological characteristics, soil water consumption, tomato physiology

## Abstract

In the cold zone of China, winter is cold and long and has a short duration of sunshine. Unheated earthen‐wall solar greenhouses are used for tomato production in winter in this region. This was an experimental investigation of different organic mulching materials (newspaper, bran, and grass) on the soil temperature, soil moisture, tomato yield, fruit quality, and water use efficiency. Organic mulching variously improved soil temperature, soil moisture, water use efficiency, and tomato yield, which is very important for greenhouse winter cultivation in this cold zone. Organic mulching regulated the soil temperature, with daily soil temperature ranges of bran, newspaper, and grass treatments being 1.6, 1.9, and 2.1°C lower than for bare land, respectively. Compared to bare land, newspaper mulching had little effect on soil temperature and fruit quality, but increased soil moisture (14.1%) and water use efficiency (WUE: WUE_*b*_, 31.3%; WUE_*y*_, 30.6%), and greatly increased yield (81.8%) and biomass (82.7%); bran mulching greatly increased soil temperature, moisture (16%), and WUE (WUE_*b*_, 60.1%; WUE_*y*_, 44.3%) and increased biomass (30.2%) and yield (17.3%); grass mulching greatly increased soil temperature and moisture (20.9%) and increased biomass (17.9%), yield (11.2%), and WUE (WUE_*b*_, 20.5%; WUE_*y*_, 13.6%). In addition, organic mulching had a good water retention effect on soil layer above 30 cm. The total soil water consumption during tomato growth was in the following order: newspaper (103 mm) > bare (74 mm) > grass (73 mm) > bran (60 mm). Soil water consumption mainly occurred in the 0‐ to 10‐cm soil layers.

## Graphical abstract

The organic mulching variously improved soil temperature, soil moisture, water use efficiency, tomato yield, and tomato fruit quality, which is very important for greenhouse winter cultivation in this cold zone. Organic mulching regulated the soil temperature, with daily soil temperature ranges of bran, newspaper, and grass treatments being 1.6, 1.94, and 2.12°C lower than for bare land, respectively. The total soil water consumption during tomato growth was in the following order: newspaper (103.0 mm) > bare (74.0 mm) > grass (72.5 mm) > bran (60.2 mm). Bran mulching was recommended for farmers in the cold and arid regions for greenhouse cultivation and even field cultivation, which is not only effective in improving utilization of scarce water resources but also increases the soil temperature.

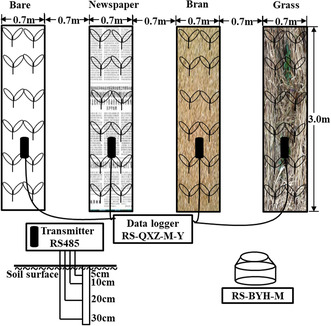

## 1 INTRODUCTION

Tomato (*Solanum lycopersicum* L.) is one of the most important vegetable plants in the world. Global production exceeds 180 million metric tons, with China and the USA as the leading producers in 2019, and production in China accounts for 20% of global output (FAOSTAT, 2021). Tomato is consumed fresh, cooked, or after processing by canning, or making juice, pulp, paste, or a variety of sauces (Zhang et al., 2016). Night temperature is the critical factor for tomato fruit setting, with the optimum range of 15–20°C. Fruit set is reduced markedly when average maximum temperature goes above 32°C and average minimum temperature goes above 21°C (Garg & Cheema, 2011). Wang et al. (2020) reported that daily water consumption ranged from 71.6 × 10^−3^ to 91.9 × 10^−3^ mm plant^−1^ under greenhouse soil cultivation.

In the cold zone (Beijing, Tianjin, Hebei, Shandong, Henan, Anhui, Shaanxi, Ningxia, southern Liaoning, east‐central Gansu, southern Xinjiang, and southern Xizang) of China, winter is cold and long and with short durations of sunshine. The average temperature in January is between −1 and 5°C, the minimum temperature occasionally drops to −30°C, and the daily temperature range is 10–15°C (Wang, 2015). Greenhouses are the only facilities that allow mass production of vegetables in winter (An et al., 2022). Various engineering and horticultural measures are employed to provide an appropriate hydrothermal environment for vegetable growth in greenhouses in cold winter.

Engineering measures are mainly heating systems, such as hot water boilers, hot air furnaces, electric heaters, and heat pumps (Mellalou et al., 2021). However, added heating systems commensurately increase the capital cost and operating costs of greenhouses, and increase the release of greenhouse gases (Chen et al., 2020). Unheated, single‐slope solar greenhouses with a north wall (insulated and with high levels of sunlight) are mostly used to improve vegetable production in the northern part of China (Tong & Christopher, 2009; Wang et al., 2010). However, solar greenhouses are enclosed and are fragile production environments in winter due to it being colder outside with negligible ventilation. Soil evaporation and crop transpiration are the main pathways of water vapor and energy exchange between crops, soil, and atmosphere in greenhouses, which directly affect the hydrothermal and energy balance of soil and atmosphere inside greenhouses. In this fragile equilibrium environment, the change of one factor (especially soil) will inevitably lead to changes in other factors, which further affect crop traits (Li et al., 2013). The soil moisture influences the change in soil temperature, with higher soil moisture, there is less variation in soil temperature, and vice versa (Kader, Senge, Mojid, & Ito, 2017).

Mulching is the most efficient measure to regulate soil moisture and temperature (Kader et al., 2019), and plastic film mulches were widely used in greenhouses during winter in the cold zone of China. However, most studies have reported that the residual plastic film accumulated in the soil damages the soil structure (Hu et al., 2020), soil physics (Wang et al., 2020), and soil hydraulic properties (Jiang et al., 2015). Meanwhile, more irrigation water or rainwater is retained in the topsoil layer due to the water‐resistant layer formed by the contribution of residual plastic film, causing higher soil evaporation and shrinkage cracks in no‐mulch soil surface (Wan et al., 2019). Compared with plastic film mulches, organic material mulching has such advantages as accessibility (Wang et al., 2015), economy (Li et al., 2003), and environmental friendliness (Yin et al., 2016). In addition, some research has reported beneficial effects of organic mulching on soil temperature and moisture and crop yield. For example, Komariah et al. (2008) considered that organic mulches reduce heat conduction into the surface soil by retaining incoming solar radiation. McMillen (2013) reported that soil moisture at a depth of 5–10 cm for wheat straw, grass clippings, and leaf debris mulching treatments was 10% higher than for bare soil. Kader, Senge, Mojid, and Nakamura (2017) mentioned that organic mulching had profound effects on microclimate, agronomic productivity, and yield of crops.

According to the above description, greenhouse cultivation is refined and sustainable agriculture, and proper cultivation management and techniques are very important, especially in the winter of cold zone of China. Meanwhile, selecting suitable mulching materials and methods to mulch the soil surface is crucial to cultivation in an unheated greenhouse when the daily average temperature is below zero. Refractory thick plastic film has been widely used in this area. To our knowledge, there are no previously published records and practice focusing on organic mulching materials for this cold environment. It is envisaged that application of organic mulching and different organic mulches would modify the water uptake and physiological characteristics of tomato plants compared with traditional planting. Field investigation is needed to explore the potential of this mulching system and different organic mulches, to enhance soil moisture availability, control soil temperature, and improve tomato physiological characteristics. The specific objectives of the study were to evaluate and compare soil temperature, soil moisture, soil water consumption, soil moisture extraction pattern (SMEP), water use efficiency (WUE), and tomato physiological characteristics of four mulching treatments (bare, newspaper, bran, and grass) under unheated greenhouse cultivation during winter of the cold zone of China.

## 2 MATERIALS AND METHODS

### 2.1 Experimental site

**FIGURE 1:**
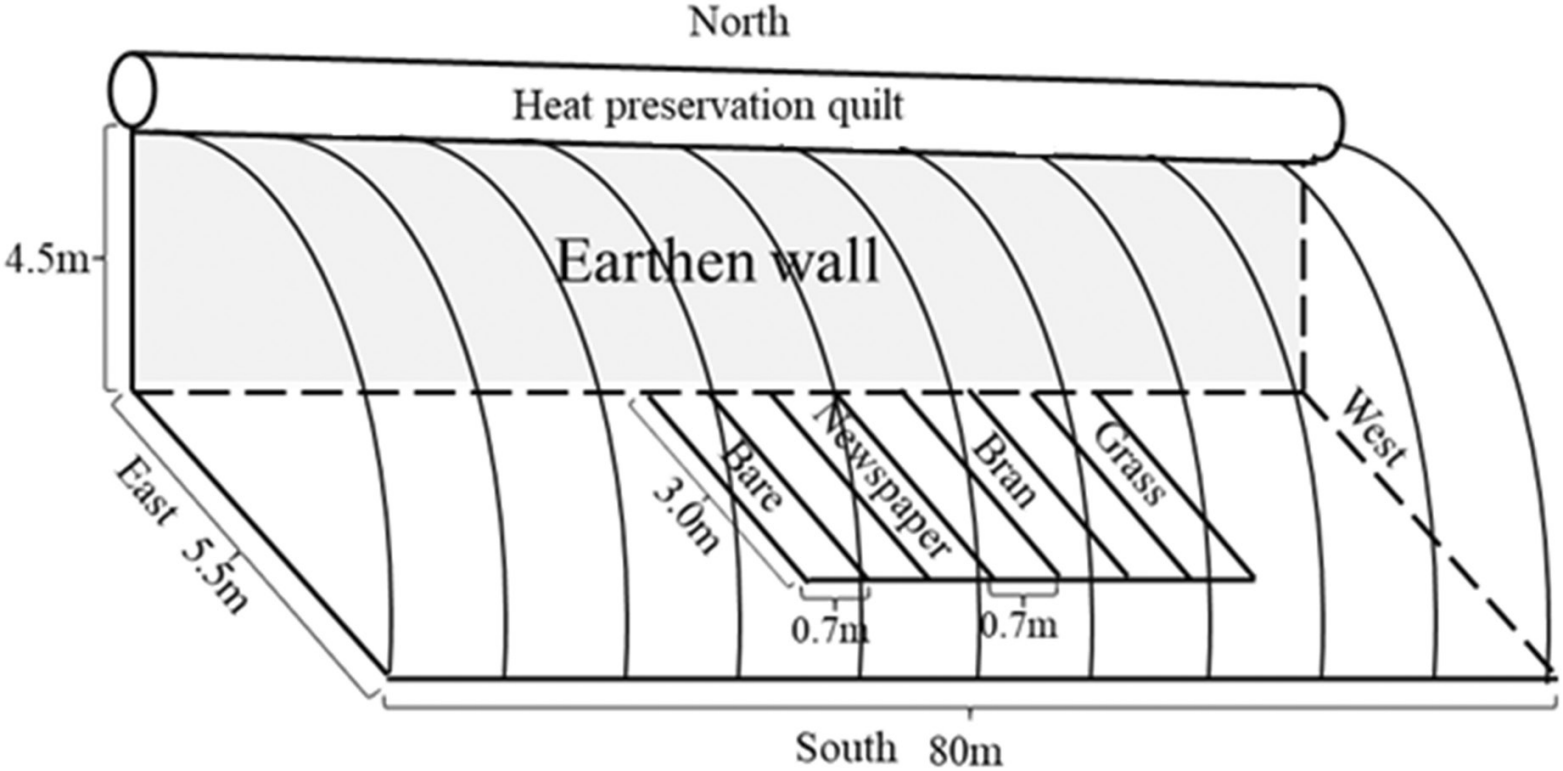
Cross‐section of an earthen‐wall unheated solar greenhouse.

**FIGURE 2:**
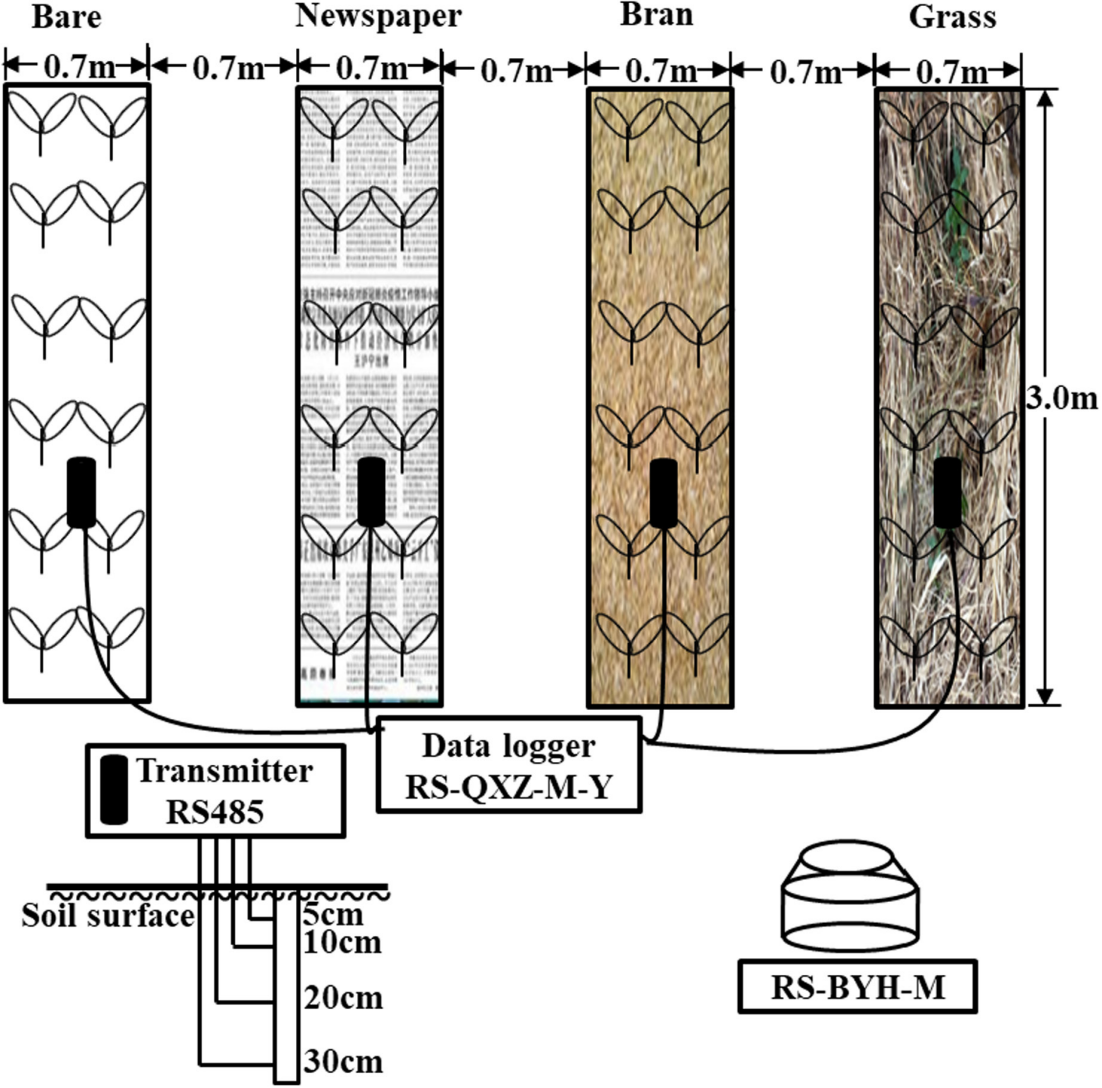
Field layout of the experiment and setup of various devices in the greenhouse.

The experiment was conducted in an earthen‐wall single‐slope solar greenhouse (80 × 5.5 m, length and width) located in Yuci County, Shanxi Province, China (37°45′53″ N, 112°46′44″ E). Figure 1 shows the greenhouse cross‐section. Heat preservation quilts were unrolled after sunset and rolled up after sunrise to preserve the inside temperature in the cold season. The experimental period was from 11 October 2020 to 23 February 2021. The temperature, relative humidity, and solar radiation inside and outside the greenhouse were measured by standard meteorological screen (RS‐BYH‐M, LvBo Instrument, Co. Ltd; Figure 2) for 10‐s intervals during the experimental period.

### 2.2 Experimental design and treatments

In the experiment, the middle position of greenhouse was selected to avoid errors due to environmental difference among plots. In order to reduce the effect of freezing plants far from earth wall in winter, the experiment used the plot near the earth wall (Figure 1). Four plots were used with different organic mulching treatments. The large plot size for each mulching treatment was 2.1 m^2^ (3.0 × 0.7 m) with a buffer zone of 0.7 m surrounding the treatments. To prevent the irrigation water from spilling out, 15 cm ridges were built around the plot. Each large plot was divided into three small plots (1.0 × 0.7 m). A no‐mulching treatment was used as control (bare). The organic mulch treatments included newspaper (0.23 kg m^−2^), wheat bran (2.8 kg m^−2^), and grass (1.5 kg m^−2^; Table 1). The thickness of mulching in this experiment follows: newspaper (0.05 cm), wheat bran (5 cm), and grass (10 cm).

**TABLE 1:** The hydrological characteristics of organic mulching material. Initial permeability rate (IPR) is the permeability rate before steady permeability.

Mulching material	Weight (kg m^−2^)	Thickness (cm)	IPR (mm s^−1^)		SPR (mm s^−1^)		NWC (%)		MWR (%)		AWR (kg m^−2^)		SWL (g kg^−1^ h^−1^)	
Bare	—	—	1.0 × 10^−4^	a	4.2 × 10^−5^	a	—		—		—		—	
Newspaper	0.23	0.05	1.2 × 10^−4^	a	6.2 × 10^−5^	a	5.7	b	364	a	0.83	b	137	a
Bran	2.8	5	1.1 × 10^−4^	a	5.3 × 10^−5^	a	24.5	ab	236	b	6.7	a	35.2	c
Grass	1.5	10	1.7 × 10^−4^	a	5.4 × 10^−5^	a	38.8	a	410	a	6.3	a	91.3	b

*Note*: Steady permeability rate (SPR) is the permeability rate after steady permeability. Natural water content (NWC) is the natural water content of mulching material. MWR is the maximum water retention of mulching material. Actual water retention (AWR) is the water retention of mulching material after irrigation. Saturated water loss (SWL) is the water loss rate of mulching material after adequate water absorption. Values followed by different lowercase letters (a–c) in the same column significantly differ (*p* < .05) based on Duncan's test.

The permeability, water retention, and water loss of each treatment were measured before experiment (Table 1). The permeability rate of each treatment was measured by double ring infiltrometer (Touma & Albergel, 1992). The double rings were driven vertically into the ground to a depth of 10 cm, and the organic mulching material was then spread into the double rings. Ten centimeters of water was injected into the outer and the inner ring simultaneously, and subsequent injections were required to meet the benchmark of 10 cm. Then, 500 mL of water was added to the inner ring, and the time required for water infiltration to 10 cm was recorded. After repeating the process multiple times, water infiltration in the inner ring gradually slowed and began to stabilize. The water infiltration rate was calculated with the following formula:

(1) $$V=\frac{10Q_{n}}{ST_{n}}$$

where *V* delineates the water infiltration rate for a certain duration (mm s^−1^). *Q*_*n*_ refers to the cumulative amount of water injected within the *n*th measurement time (mL). *S* indicates the infiltration area of the inner ring (mm^2^). *T*_*n*_ is the interval among the *n*th measurements (s). The result showed that the initial (permeability rate before steady permeability) and steady (permeability rate after steady permeability) permeability rates among treatments did not significantly differ (Table 1).

**FIGURE 3:**
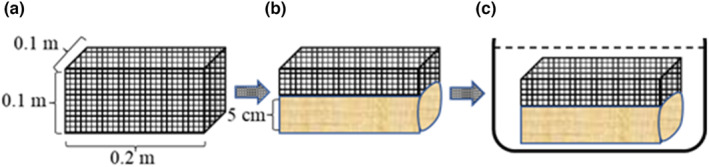
The schematic diagram of water retention and saturated water loss (a is a special diamond mesh cage; b is the schematic of the 5‐cm bran; c is the schematic of the cage fitted with organic mulch immersed in water).

The water retention and saturated water loss of each mulching material were measured by the soaking–extracting method (Dong et al., 2021). The naturally air‐dried organic mulches were placed in a special diamond mesh (Mesh diameter 0.5 mm) cage (0.2 × 0.1 × 0.1 m; Figure 3) Then, the diamond mesh cage was completely soaked in water for 24 h, suspended (air drying) for 24 h, and weighed. The calculation formulas are as follows:

(2) $$\mathrm{NWC}=\left(\frac{G_{n}-G_{d}}{G_{d}}\right)\times 100\%$$

(3) $$\mathrm{MWR}=\left(\frac{G_{a24}-G_{n}}{G_{n}}\right)\times 100\%$$

(4) $$\mathrm{AWR}=\left(G_{a24}-G_{n}\right)/S_{m}$$

(5) $$\mathrm{SWL}=\frac{G_{a24}-G_{l24}}{G_{n}}\times 1000/24$$

where NWC is the natural water content of mulching material (%), MWR is the maximum water retention of mulching material (%), AWR is the water retention of mulching material after irrigation (kg m^−2^), SWL is the water loss rate of mulching material after adequate water absorption (g kg^−1^ h^−1^), *G*_*n*_ is the weight of the natural mulching material (kg), *G*_*d*_ is the weight of mulching material after drying (kg), *G*_*a*24_ is the weight of mulching material after soaking for 24 h (kg), *G*_*l*24_ is the weight of mulching material after water loss for 24 h (kg), and *S*_*m*_ is an area of natural mulching material (m^2^).

The water content of natural organic mulching material was in the following order: grass (38.8%) > bran (24.5%) > newspaper (5.7%). The grass had the highest maximum water retention (410%), followed by newspaper (364%) and bran (236%) after soaking for 24 h. The highest actual water retention after adequate irrigation was for bran treatment (6.7 kg m^−2^), followed by grass (6.3 kg m^−2^) and newspaper (0.83 kg m^−2^). During the 24‐h water loss experiment, the organic mulching water loss rate after adequate water absorption (soaking for 24 h) was in the following order: newspaper (137 g kg^−1^ h^−1^) > grass (91.3 g kg^−1^ h^−1^) > bran (35.2 g kg^−1^ h^−1^).

Fruit tomato (*Solanum lycopersicum* ‘Provence’) plants were the experimental variety. The seedlings were cultivated at a seed company. When reaching a height of approximately 20 cm, they were transplanted to the plots in a randomized complete block design with 12 plants per treatment. The planting and row spacings were both 0.5 m. Lateral buds were pruned as they sprouted, and the top bud was pruned in the seventh week (49–56 days after transplanting) when the plant was in the third flower‐cluster stage.

### 2.3 Soil environment

The depth of the main root zone of the tomato crop was <30 cm from field observation. Undisturbed soil samples were collected from the tomato root zone: 0–10, 10–20, and 20–30 cm soil profile under bare land treatment. The soil texture of the study site was silty loam; soil bulk density was 1.4 g cm^−3^ in the 0‐ to 30‐cm soil layer. The field capacity and permanent wilting point moisture contents were 0.36 and 0.14 cm^3^ cm^−3^, respectively.

The soil temperature and absolute soil moisture content were measured at four depths (5, 10, 20, and 30 cm) under each mulching treatment at 10‐s intervals by a soil temperature and moisture transmitter (RS485, LvBo Instrument, Co. Ltd). The data were collected by a datalogger (RS‐QXZ‐M‐Y, LvBo Instrument, Co. Ltd; Figure 2). The soil water consumption (SWC, mm), defined as the cumulative soil water reduction (${\mathrm{SWC}}_{i,j}$) in the root zone (0–30 cm) between two consecutive irrigations throughout the tomato growing period, was estimated. The ${\mathrm{SWC}}_{l,i}$ (mm) was estimated from the summation of soil moisture decrease in 0–10, 10–20, and 20–30 cm soil layers (*l* = 1–m) between two consecutive irrigation events during the tomato growing period (*i* = 1–n) as follows:

(6) $$\mathrm{SWC}=\sum_{i=1}^{n}\left(\sum_{l=1}^{m}{\mathrm{SWC}}_{l,i}\right)=\sum_{i=1}^{n}\left[\sum_{l=1}^{m}\left({Ɵ}_{\mathrm{IA},l,i}-{Ɵ}_{\mathrm{IB},l,i+1}\right)\times h_{l}\right]$$

where, $\;\theta_{\mathrm{IB},l,i}$ (cm^3^ cm^−3^) is the soil moisture content of the *l*‐th soil layer just before the *i*‐th irrigation, $\;\theta_{\mathrm{IA},\mathrm{li}}$ (cm^3^ cm^−3^) is the soil moisture content of the *l*‐th soil layer just after the *i*‐th irrigation, $h_{l}$ (mm) is the thickness of the *l*‐th soil layer, m is the number of the soil layer, and n is the number of irrigations (Kader, Senge, Mojid, & Nakamura, 2017).

The SMEP was defined as the ratio of soil moisture reduction in the *l‐*th layer to the total soil moisture reduction within all the soil layers. The *l‐*th layer of SMEP_*l*_ was calculated as follows:

(7) $${\text{SMEP}}_{l}=\frac{{Ɵ}_{l}∙h_{l}}{\sum_{l=1}^{n}{Ɵ}_{l}∙h_{l}}∙100$$

where $h_{l}$ (cm) is the thickness of the *l*‐th soil layer and $\;\theta_{l}$ (cm^3^ cm^−3^) is the soil moisture content of the *l*‐th soil layer (Kader, Senge, Mojid, Onishi, & Ito, 2017).

### 2.4 Agronomic, physiological, and food chemistry management

Spray irrigation (simulated rainfall) was carried out randomly when plant dehydration or soil drying was observed.

Relative leaf chlorophyll (soil–plant analysis development: SPAD values) and relative leaf nitrogen (N) content (mg g^−1^) were measured once a week using a plant nutrition meter (LYS‐4N, LvBo Instrument, Co. Ltd). There is an exact linear relationship between SPAD values and extracted leaf chlorophyll (Markwell et al., 1995; Yadava, 1986); therefore, this is used to represent leaf chlorophyll in this study.

Red fruits were harvested once a week during harvest season. The yield (g plant^−1^) on each plant was measured during the harvest season.

The WUE was calculated according to biomass (WUE_*b*_, g mm^−1^: grams of biomass produced per mm of irrigation water) and yield (WUE_*y*_, g mm^−1^: grams of yield produced per mm of irrigation water).

(8) $${\mathrm{WUE}}_{b}=\frac{\text{Biomass}}{\text{Water consumption}}$$

(9) $${\mathrm{WUE}}_{y}=\frac{\text{Yield}}{\text{Water consumption}}$$

Fully mature tomato fruits were used for the fruit quality measurements. Twelve fruits were chosen for each treatment, making a total of 48 fruits for the measurements. Fruit firmness was evaluated with a penetrometer (GY‐4, LvBo Instrument, Co. Ltd). Each fruit was juiced using a small juicer, and the fruit sugar content (%) and fruit acid content (%) were measured with a Pocket brix‐acid meter (PAL‐BX/ACID1, ATAGO, Co. Ltd). In addition, taste index was calculated using the equation of Hernández‐Suárez et al. (2008):

(10) $$\text{Taste index}=\frac{\text{Sugar content}}{20\times \text{Acidity}}+\text{Acidity}$$

### 2.5 Data analysis

Statistical analysis was performed using one‐way analysis of variance, and the results were compared using Duncan's test with a confidence level of 5% using the R programming language.

## 3 RESULTS

### 3.1 Meteorological parameters

**FIGURE 4:**
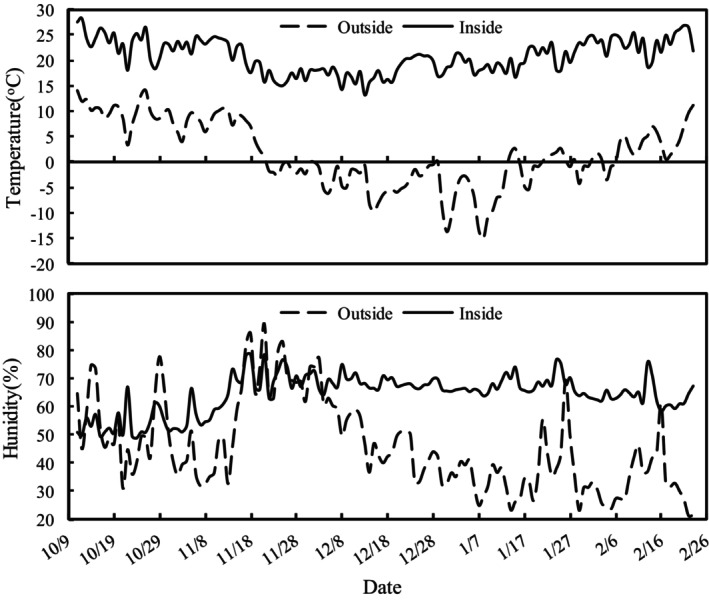
Daily mean temperature and humidity inside and outside the greenhouse during the crop growing period (11 October 2020 to 24 February 2021).

Inside the greenhouse, the mean monthly air temperature during the experimental period first decreased from 23.6°C in October to 17.9°C in December, then increased to 23.7°C in February (Figure 4); the average temperature was 20.8°C. The mean monthly relative humidity first increased and then decreased (Figure 4); the average relative humidity was 64.4%. The mean monthly solar radiation first decreased from 13.0 MJ m^−2^ day^−1^ in October to 7.5 MJ m^−2^ day^−1^ in December, then increased to 13.0 MJ m^−2^ day^−1^ in February; and the average solar radiation was 10.3 MJ m^−2^ day^−1^.

Outside the greenhouse, the mean monthly air temperature during the experimental period first decreased from 10.3°C in October to −4.1°C in December, then increased to 3.4°C in February (Figure 4); the average temperature was 1.5°C. The mean monthly relative humidity decreased (Figure 4) and the average relative humidity was 45.9%. The mean monthly solar radiation first decreased from 17.6 MJ m^−2^ day^−1^ in October to 15.8 MJ m^−2^ day^−1^ in December, then increased to 20.1 MJ m^−2^ day^−1^ in February; and the average solar radiation was 17.4 MJ m^−2^ day^−1^.

### 3.2 Soil temperature

**FIGURE 5:**
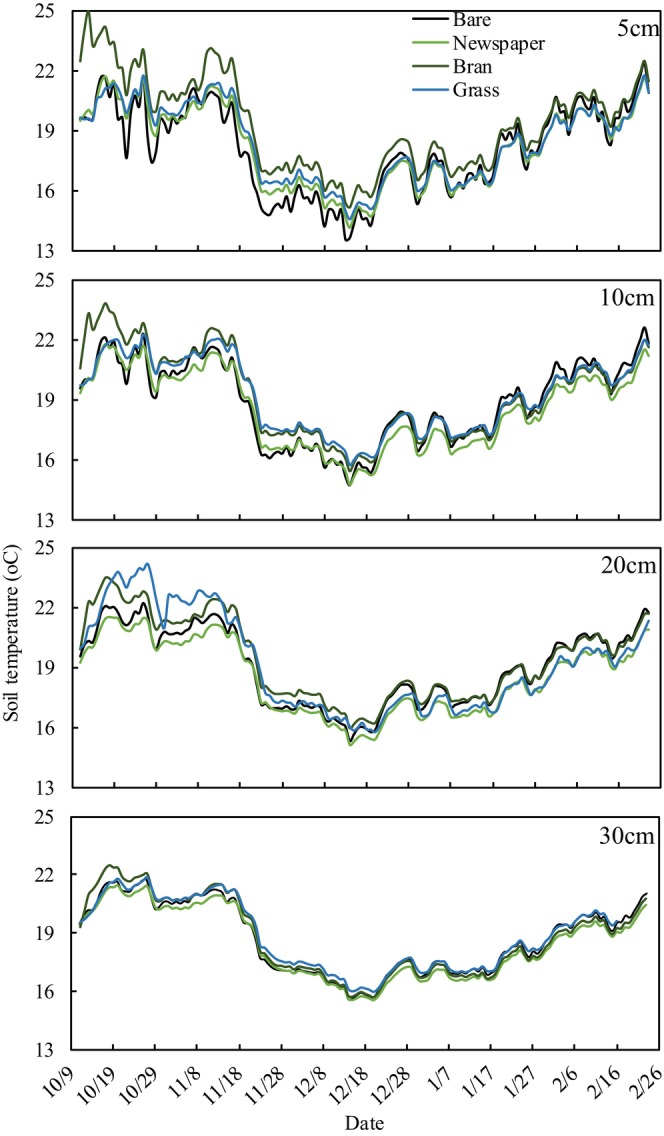
Daily mean soil temperature (°C) in the experimental tomato plots under various mulching treatments during the crop growing period.

**FIGURE 6:**
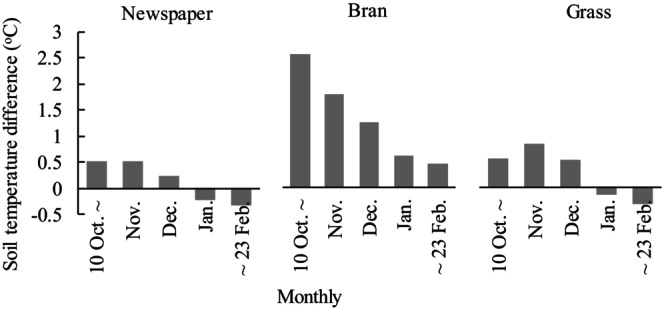
Monthly mean temperature difference of 5‐cm depth soil between organic mulching treatments and bare land during the growing period (October 2020 to February 2021).

Table 2 shows that the daily minimum soil temperature, daily mean soil temperature (DMT), daily maximum soil temperature, and daily soil temperature range were over the 0–30 cm soil profile during the growing period under four different treatments. The daily minimum soil temperatures of mulching treatments (newspaper, bran, and grass) were significantly higher than the bare treatment. The daily maximum soil temperature of mulching treatments was significantly lower than the bare treatment. The daily soil temperature range of bare treatment was significantly higher than in mulching treatments, with the following order: bare (3.4°C) > bran (1.9°C) > newspaper (1.5°C) > grass (1.3°C). The DMT of bran treatment (19.2°C) was significantly higher than grass treatment (18.9°C), followed by bare (18.7°C), and newspaper (18.4°C) treatments. For bran mulching treatment, the DMT at 5‐cm depth was 1.3°C higher than for bare treatment, followed by 10 cm (0.43°C), 20 cm (0.42°C), and 30 cm (0.02°C; Table 3, Figure 5). For grass mulching treatment, the DMT at 10‐cm depth was 0.32°C higher than for bare treatment, followed by 5 cm (0.31°C), 20 cm (0.17°C), and 30 cm (0.06°C). However, for newspaper mulching treatment, the DMT only at 5‐cm depth was 0.15°C higher than for bare treatment; in other soil layers temperatures were 0.3–0.54°C lower than for bare treatment (Figure 5). The difference in DMT at a 5‐cm depth between the bare and mulching treatments (newspaper, bran, and grass) decreased through the tomato growth period (from October 2020 to February 2021); notably, newspaper and grass treatments had lower temperatures than the bare treatment from January (Figure 6).

**TABLE 2:** The daily minimum soil temperature (DMnT), daily mean soil temperature (DMT), daily maximum soil temperature (DMxT), daily soil temperature range (DTR), and daily mean soil moisture (DMSM) over the 0‐ to 30‐cm soil profile during the growing period under four different treatments.

Treatment	DMnT (°C)		DMT (°C)		DMxT (°C)		DTR (°C)		DMSM (%)	
Bare	17.2 ± 2.4	c	18.7 ± 2.0	b	20.7 ± 2.9	a	3.4 ± 3.2	a	18.8 ± 3.9	c
Newspaper	17.7 ± 2.0	b	18.4 ± 1.9	c	19.2 ± 2.1	d	1.5 ± 1.3	c	21.4 ± 2.4	b
Bran	18.3 ± 2.2	a	19.2 ± 2.1	a	20.2 ± 2.5	b	1.9 ± 1.7	b	21.8 ± 3.2	b
Grass	18.2 ± 2.1	a	18.9 ± 2.0	b	19.6 ± 2.1	c	1.3 ± 1.0	c	22.7 ± 2.9	a

*Note*: Data are the means ± standard deviations. Values followed by different lowercase letters (a–d) in the same column indicate significant differences (Duncan's test, *p* < .05) among the four treatments.

**TABLE 3:** The daily mean soil temperature and daily mean soil moisture at four depths (5, 10, 20, and 30 cm) in the experimental tomato plots under various mulching treatments.

Treatments	Daily mean soil temperature (°C)								Daily mean soil moisture (%)							
	5 cm		10 cm		20 cm		30 cm		5 cm		10 cm		20 cm		30 cm	
Bare	18.1 ± 2.2	b	18.9 ± 2.0	ab	19.0 ± 1.9	a	18.7 ± 1.8	a	13.2 ± 2.4	d	19.2 ± 1.9	c	21.2 ± 2.0	c	21.48 ± 1.9	b
Newspaper	18.2 ± 2.0	b	18.5 ± 1.9	b	18.5 ± 1.9	a	18.4 ± 1.8	a	19.2 ± 2.4	b	21.4 ± 2.0	b	22.3 ± 1.5	b	22.79 ± 1.6	c
Bran	19.4 ± 2.3	a	19.3 ± 2.2	a	19.4 ± 2.1	b	18.7 ± 2.0	a	17.4 ± 2.6	c	22.5 ± 1.6	a	22.9 ± 1.7	a	24.23 ± 0.7	b
Grass	18.4 ± 1.9	b	19.2 ± 1.8	a	19.2 ± 2.4	a	18.8 ± 1.8	a	21.7 ± 4.1	a	21.7 ± 2.3	b	22.4 ± 1.9	b	25.29 ± 0.9	a

*Note*: Data are the means ± standard deviations. Values followed by different lowercase letters (a–d) in the same column indicate significant differences (Duncan's test, *p* < .05) among the four treatments.

### 3.3 Soil moisture and SWC

**FIGURE 7:**
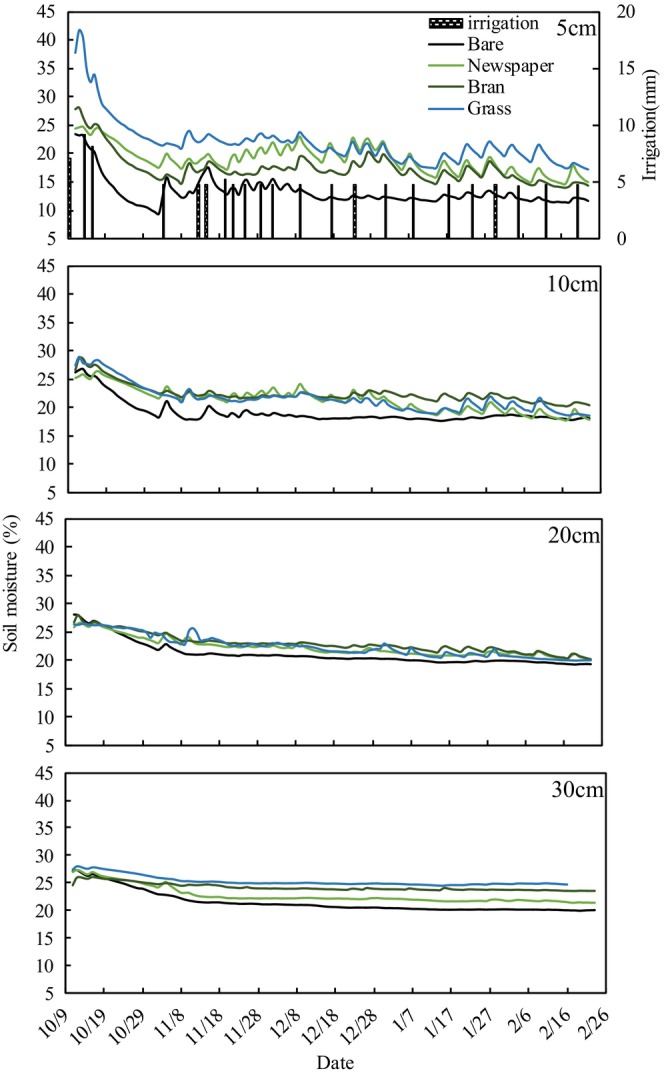
Daily mean soil moisture (%) in the experimental tomato plots under various mulching treatments along with irrigation during the crop growing period.

The soil moisture content in the tomato plots under the mulching treatments (newspaper, bran, and grass) fluctuated with irrigation events (Figure 7). The daily mean soil moisture (DMSM) of bare treatment was significantly lower than mulching treatments (Table 2), which was in the following order: bare (18.8%) < newspaper (21.4%) < bran (21.8%) < grass (22.7%). For grass mulching treatment (Table 3, Figure 7), the DMSM content at a 5‐cm depth was 8.5% higher than for bare treatment, followed by 30 cm (3.8%), 10 cm (2.5%), and 20 cm (1.2%). For bran mulching treatment, the DMSM content at 5‐cm depth was 4.2% higher than for bare treatment, followed by 10 cm (3.3%), 30 cm (2.8%), and 20 cm (1.7%). For newspaper mulching treatment, the DMSM content at 5‐cm depth was 5.94% higher than for bare treatment, followed by 10 cm (2.2%), 30 cm (1.3%), and 20 cm (1.1%).

**FIGURE 8:**
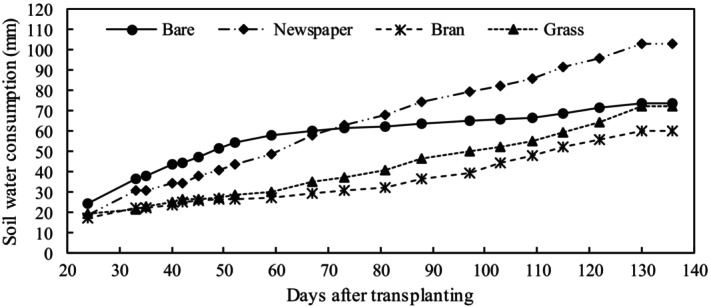
Cumulative soil water consumption from the 0‐ to 30‐cm soil profile throughout the tomato growing season under four different treatments.

The SWC of mulch treatments (newspaper, bran, and grass) during the tomato growth period showed that the SWC was low in the early part of the growth season (~60 DAT, days after transplants), but increased over time until tomato maturity (Figure 8). Conversely, SWC of the bare treatment was high in the early part of the growth season (~60 DAT) but then decreased. The total SWC during tomato growth was in the following order: newspaper (103 mm) > bare (74 mm) > grass (73 mm) > bran (60 mm). However, in the earlier growth season (~60 DAT), SWC was in the following order: bare (58 mm) > newspaper (48 mm) > grass (30 mm) > bran (27 mm).

### 3.4 SMEP

The SMEP among the treatments is shown in Table 4. For each treatment, the SMEP at 5‐cm depth was significantly higher than for other soil layers, and decreased with increasing soil depth except for the bran treatment. The SMEP under bare, newspaper, and grass treatments did not significantly differ except at 10‐cm depth where SMEP was slightly lower for bare than the other treatments. The bran treatment at 5‐cm depth had significantly lower SMEP than other treatments. The SMEP for the 0‐ to 10‐cm soil layer was in the following order: grass (78.1%) > newspaper (77%) > bare (76.6%) > bran (60%).

**TABLE 4:** Soil moisture extraction pattern at 5‐, 10‐, 20‐, and 30‐cm depths over the 0‐ to 30‐cm soil profile during the growing period under four different treatments.

Depth	Bare (%)			Newspaper (%)			Bran (%)			Grass (%)		
5 cm	60.3 ± 13.8	a	A	52.5 ± 14.8	a	AB	41 ± 14.5	a	B	55.4 ± 14.6	a	A
10 cm	16.3 ± 6.5	b	B	24.5 ± 9.7	b	A	19 ± 7.9	c	AB	22.7 ± 8.9	b	AB
20 cm	15.9 ± 3.9	b	B	15.9 ± 5.8	bc	B	29.7 ± 10.9	b	A	14.9 ± 5.6	bc	B
30 cm	7.5 ± 4.4	b	A	7.1 ± 3.9	c	A	10.3 ± 3.6	d	A	7 ± 3.4	c	A

*Note*: Data are the means ± standard deviations. Values followed by different lowercase letters (a–d) in the same column indicate significant differences (Duncan's test, *p* < .05) among the four depths (5, 10, 20, and 30 cm) within treatments. Values followed by different capital letters (A–B) in the same row indicate significant differences (Duncan's test, *p* < .05) at the same depths between treatments.

### 3.5 Biomass, yield, WUE, and fruit quality

Plant biomass, relative leaf chlorophyll, relative leaf N content, and yield under bare, bran, and grass treatments did not significantly differ, but these were significantly lower than newspaper treatments (Table 5). The WUEs for plant biomass and yield significantly differed among all treatments. The WUE_*b*_ and WUE_*y*_ were in the following order: bran > newspaper > grass > bare (Table 5).

**TABLE 5:** Effect of different mulching on plant biomass, relative leaf chlorophyll, relative leaf N content, yield, water use efficiency for biomass (WUE_*b*_), and water use efficiency for yield (WUE_*y*_).

Treatment	Biomass (g plant^−1^)		Relative leaf chlorophyll		Relative leaf N content (mg g^−1^)		Yield (g plant^−1^)		Water con. (mm plant^−1^)		WUE_*b*_ (g mm^−1^)		WUE_*y*_ (g mm^−1^)	
Bare	1112 ± 396	b	28.5 ± 3.7	b	11.7 ± 1.2	b	801 ± 285	b	6.2 ± 2.2	b	180 ± 0.02	d	130 ± 0.02	d
Newspaper	2031 ± 441	a	29.3 ± 3	a	11.9 ± 0.96	a	1456 ± 316	a	8.6 ± 1.9	a	237 ± 0.01	b	170 ± 0.01	b
Bran	1447 ± 345	b	28.2 ± 3.1	b	11.6 ± 0.98	b	939 ± 224	b	5.0 ± 1.2	b	289 ± 0.03	a	187 ± 0.03	a
Grass	1311 ± 576	b	28.3 ± 3.2	b	11.6 ± 1.0	b	890 ± 391	b	6.0 ± 2.7	b	217 ± 0.02	c	147 ± 0.02	c

*Note*: Data are the means ± standard deviations. Values followed by different lowercase letters (a–d) in the same column significantly differ (*p* < .05) based on Duncan's test.

The fruit sugar content under bare treatment was significantly higher than for other treatments, and bran treatment had the lowest value. The sugar content among all treatments was in the following order: bare (9.3%) > grass (8.4%) > newspaper (7.6%) > bran (7.3%; Table 6). The values of fruit acid content among all treatments showed the same order as sugar content, but differences were not significant. The bare (1.4) and grass (1.4) treatments showed the highest taste index, followed by newspaper (1.3) and bran (1.3). The fruit firmness under newspaper treatment was significantly higher than other treatments, and grass treatment had the lowest value.

**TABLE 6:** Effect of different mulching on fruit quality.

Treatment	Sugar (%)		Acid (%)		Taste index		Fruit firmness (kg)	
Bare	9.3 ± 0.97	a	0.87 ± 0.17	a	1.4 ± 0.1	a	1.3 ± 0.51	b
Newspaper	7.6 ± 0.64	bc	0.81 ± 0.18	a	1.3 ± 0.11	b	1.8 ± 0.43	a
Bran	7.3 ± 0.64	c	0.71 ± 0.15	a	1.3 ± 0.08	b	1.4 ± 0.34	b
Grass	8.4 ± 1.54	b	0.86 ± 0.29	a	1.4 ± 0.19	a	1.1 ± 0.38	b

*Note*: Data are the means ± standard deviations. Values followed by different lowercase letters (a‐c) in the same column significantly differ (*P* < .05) based on the Duncan’s test.

## 4 DISCUSSION

The mulching treatments (newspaper, bran, and grass) reduced the daily maximum soil temperature but raised the daily minimum soil temperature compared to the bare treatment. The reason might be because organic mulching (e.g., straw, grass, and wood chips) increases albedo and decreases thermal conductivity of the soil surface, reducing solar energy that penetrates the soil (Li et al., 2013), meanwhile reducing the soil energy dissipating, resulting in a significant reduction in daily soil temperature range. Mulch acts as a buffer to insulate soil from heat and cold temperatures (Kader et al., 2019). Olasantan (1999) also reported that compared to nonmulched soil, mulched soil showed higher soil temperature during colder weather and lower soil temperature during warmer weather. In addition, Wang et al. (2021) summarized that organic mulch helps moderate temperature and protect soil and roots from extreme temperatures.

For DMT, bran and newspaper treatments significantly increased and decreased soil temperature compared to bare treatment, respectively, but grass treatment had no effect on soil temperature. This may be because covering the soil surface with dense and poorly breathable bran seriously hinders the exchange of heat capacity between soil and atmosphere, thus maintaining soil temperature (Kodešova et al., 2014). However, the smoothness and lighter color of newspaper reflect sunlight (Haapala et al., 2014; Kader, Senge, Mojid, & Ito, 2017). The heat was exchanged quickly between soil covered by fluffy grass and the atmosphere, and soil heat capacity was lost more easily for grass mulching in a cooler external environment (Al‐Shammary et al., 2020).

The difference in DMT between bare and mulching treatments at 5‐cm depth (decreased through the tomato growth period, Figure 6) may be because mulching warms the topsoil quickly during the day and dissipates heat slowly at night compared to bare land under direct sunlight (Zhou et al., 2009). However, as the canopy grows, the ground receives progressively less sunlight and the heat harbored by the mulch layer does not transfer to the soil (Dai et al., 2021). In addition, organic mulch degrades on the soil surface and this reduces the action of the barrier during later stages of plant growth.

The DMSM under organic mulching treatments was significantly higher than bare treatment, and grass treatment had the highest value (20.8%), followed by bran (16%) and newspaper (14.1%). The SWC for bran and grass treatments did not significantly differ from that for bare treatment but was significantly higher for newspaper than bare treatment. This may be because mulching alters the moisture and temperature environment of the soil by affecting the hydrothermal regime (Kader, Senge, Mojid, & Ito, 2017). Each type of mulching material has a particular set of characteristics. Mulching materials affect the soil moisture environment mainly through their water retention, water permeability, and decomposability. In this study, the influence of water permeability of mulching materials on soil moisture can be ignored due to the flood irrigation method. The DMSM contents of mulching treatments were higher than for bare treatment. The reason may be that mulching physically prevents vertical evaporation from the soil surface by blocking direct sunlight reaching the soil surface, and mulching measures also reduce water runoff on the soil surface (Saglam et al., 2017), thus preserving soil water. In addition, some organic mulch acts as a sponge and retains rainfall and irrigation water, thus preventing runoff and providing water at the time of crop requirement (Iqbal et al., 2020). Zhao et al. (2014) also reported that mulching treatments stored more soil moisture compared to bare soil.

**FIGURE 9:**
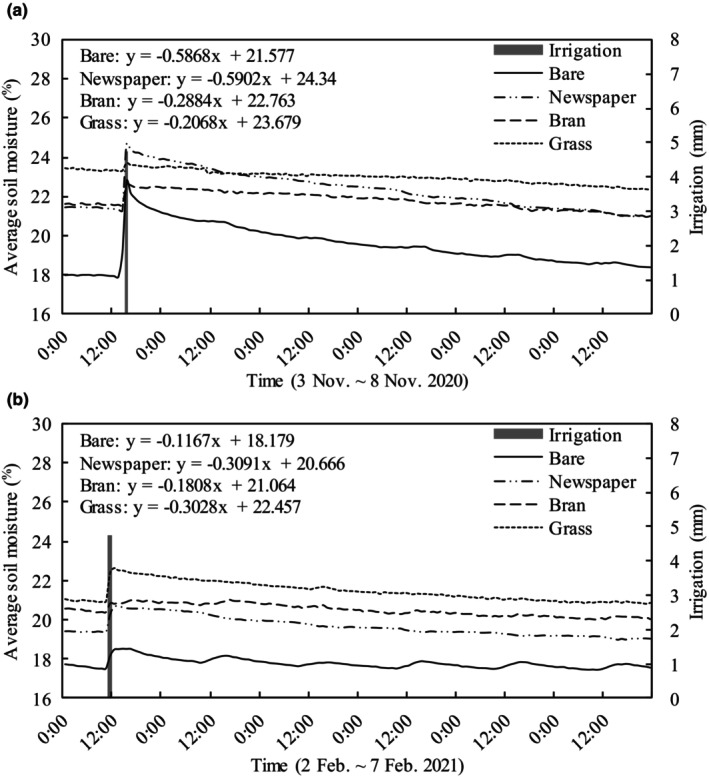
The dynamic of hourly mean soil moisture among the treatments in the earlier (3–8 November 2020, a) and later (2–7 February 2021, b) growth stages after irrigation.

Among the mulching treatments (newspaper, bran, and grass), the newspaper had the lowest DMSM and highest SWC. Newspaper mulching with its smooth surface, low porosity, negligible actual water retention (0.83 kg m^−2^), and low water loss characteristics (137 g kg^−1^ h^−1^) greatly affected the soil and resulted in high evaporation efficiency after irrigation (Haapala et al., 2014). The dynamic of hourly mean soil moisture among the treatments in the earlier (Figure 9A) and later (Figure 9B) growth stages after irrigation further substantiates these findings. Soil water under newspaper and bare treatments was rapidly lost in the earlier growth stages compared to grass and bran treatments; however, there was no difference in the rate of water loss among treatments in the later growth stages. This is because water loss at the earlier stage of plant growth is mainly by surface evaporation, yet the smooth and lighter color of the newspaper surface speeds up evaporation of irrigation water. However, with plant canopy growth, water loss is mainly dominated by plant consumption because sunlight does not reach the ground directly. In general, with plant growth and newspaper degradation, the function of newspaper on temperature preservation and water insulation gradually decreases. However, the organic matter produced by the degradation of newspapers is good fertilizer for later growth of plants. In this study, the coverage of newspaper was two layers, and the actual water retention after irrigation was only 0.83 kg m^−2^, far below that for bran (6.7 kg m^−2^) and grass (6.3 kg m^−2^). Increasing the coverage layers of newspaper would theoretically increase the soil temperature, water retention of newspaper, and organic matter production, but would reduce water permeability. Therefore, appropriate coverage of newspaper requires further experimentation.

Bran had great water retention (6.7 kg m^−2^) and slow water loss characteristics (35.2 g kg^−1^ h^−1^) due to its inherently fine porous structure, which results in irrigation water not only reaching the soil surface but also some being retained in the bran layer. As the soil water infiltrates and is absorbed by the crops, the water in bran will be slowly released into the soil and to maintain the soil moisture. In addition, when irrigation water reaches the soil surface, some infiltrates and some evaporates. Yuan et al. (2009) also reported that porous materials such as camellia shell, straw, and straw and ecological pad, and slow water vapor transmission to the atmosphere. However, evaporated water vapor reaches the mulch and then falls back to the soil as droplets (Ahmad et al., 2020). In particular, the bran layer with fine pores seriously hinders soil water evaporation. Meanwhile, compared with newspaper and grass treatments, after irrigation, the bran treatment more easily forms a crust due to temperature increase from direct sunlight, which helps to preserve the water in the bran layer as well as the soil moisture.

Grass mulching greatly increased DMSM, consistent with the conclusion that mulching increased soil moisture. However, the SWC was close to bare treatment and much higher than that of bran treatment. This may be due to fluffy grass mulch macropores that block direct sunlight reaching the soil surface, but not preventing the gas exchange between soil and atmosphere. Water contained in grass was readily transformed into vapor to satisfy evaporative demand from the soil and grass following the energy balance at the soil surface. In this process, most of the water vapor is lost through evaporation although some evaporated water vapor reaches the mulch and then falls back. However, grass mulching treatment resulted in the highest SWC during the whole growth period. This may be for two reasons, grass mulching blocks direct sunlight reaching the soil surface, resulting in reduced evaporation and a large proportion of evaporated water vapor condenses into grass, then falls back to the soil surface and evaporates again after irrigation, this repeated water cycle delays soil moisture loss until the next irrigation.

The SMEP mainly focuses on the soil layer of 0–10 cm, especially 0–5 cm. This result is consistent with the findings of Kader, Senge, Mojid, Onishi, and Ito (2017). Soil moisture in the upper surface layer (0–10 cm) was highly dynamic due to water vapor fluxes across the soil–atmosphere interface, thus increasing the evaporation loss at the surface layer (Bittellia et al., 2008), and most of the water consumed by plants is in this zone due to high root mass (Lenka et al., 2009). However, the bran treatment at 5‐cm depth showed significantly lowest SMEP among treatments. This is because the thickness of bran cover was about 5 cm, and the soft, thick layer of small bran particles had poor porosity, thus hindering the water vapor exchange between atmosphere and soil surface. Especially, irrigation water filled the pores between bran particles (high water retention) after irrigation, which almost cut off the circulation channel between soil and atmosphere, and so reducing the surface soil moisture evaporation loss. In addition, crusting of the bran layer surface further seriously affected water vapor circulation between soil surface and atmosphere.

Plant growth is a very complex process, and is affected by many factors such as external environment (e.g., soil, water, and climate; Chakma et al., 2021), plant differences (e.g., variety, disease resistance, and growth phenology; Dudas, Kotroczo, et al., 2017; Dudas, Szalai, et al., 2017), and cultivation environment (e.g., field cultivation, greenhouse cultivation, and hydroponics; Wang et al., 2020; Zhang et al., 2016). In this study, the tomato biomass, relative leaf chlorophyll, relative leaf N content, and yield under newspaper treatment were significantly higher than the other treatments. This may be because the smoothness and lighter color of the newspaper surface increased the reflection of solar radiation. The reflection of light from newspaper accelerates photosynthesis in plants under conditions of severe lack of light (single‐slope solar greenhouse with short sunshine time and thick film). Secondly, compared to other treatments, newspaper mulch slowed the infiltration of flood irrigation and so reduced the leaching of soil nutrients by irrigation water (Karhu et al., 2021) and maintained the soil nutrients to provide nutrients for tomato growth. In addition, the organic mulches (newspaper, bran, and grass) decompose and add nutrients to soil. The additional nutrients plausibly contribute to increase crop yield compared to bare soil. A similar result was reported by Kader, Senge, Mojid, and Ito (2017). The WUE results for biomass and yield indicated that mulching was very effective in saving water and increasing the yield of tomato. These results were similar to those of Yin et al. (2015).

Tomato fruit quality is susceptible to various biotic and abiotic stresses, with drought and salinity being major abiotic stresses that can positively affect fruit quality (Chakma et al., 2021). In this study, the bare treatment had the highest fruit quality, possibly due to the lowest root zone moisture during the fruit ripening period. Water deficit decreases the photo‐assimilate dilution by decreasing the water level within fruits, thus promoting the accumulation of assimilates within fruits and improving quality parameters (Chakma et al., 2021; Kuscu et al., 2014). In addition, root zone temperature influences transport from source to sink during fruit development and the biochemical transformation during fruit ripening. High temperature increases the fruit's sugar content while decreasing the acid content (Mesa et al., 2022). Meanwhile, large diurnal temperature difference increases tomato fruit's sugar contents while organic acid contents are diminished (Ruiz‐Nieves et al., 2020). In this study, the bare treatment had highest maximum temperature in the root zone and largest diurnal temperature difference. The superimposed effects of the above multiple factors promoted the best fruit quality in the bare treatment.

## 5 CONCLUSION

Overall, organic mulching tended to improve soil temperature, soil moisture, WUE, and tomato yield, which is very important for greenhouse cultivation in winter in the cold zone of China. In particular, the water retention of organic mulching was clear, and this influence extended to a depth of 30 cm.

Based on the study results, the three organic mulching materials had advantages and disadvantages. Newspaper mulching showed poor soil temperature and water protection, good biomass and yield, and good fruit quality. Bran mulching showed good soil temperature and water protection, middle biomass and yield, and poor fruit quality. Grass mulching had middle soil temperature and water protection, poor biomass and yield, and middle fruit quality. Newspaper mulching had no effect on tomato fruit quality, and bran and grass mulching reduced fruit quality. In addition, mulching regulated the soil temperature; the daily soil temperature ranges of bran, newspaper, and grass were 1.6, 1.9, and 2.1°C lower than for bare land, respectively.

However, the amount of organic mulch used in this study may not have reached the necessary threshold, and further research is needed to determine the threshold of organic mulch suitable for greenhouse cultivation in the cold zone in winter.

## AUTHOR CONTRIBUTIONS

**Pengfei Zhang:** Conceptualization (lead); writing – original draft (lead); writing – review and editing (lead). **Zhaorui Zhang:** Writing – original draft (equal). **Menglin Xiao:** Writing – original draft (equal). **Jinlong Chao:** Conceptualization (equal); writing – review and editing (equal). **Yanyan Dai:** Writing – original draft (equal); writing – review and editing (equal). **Geng Liu:** Conceptualization (equal); writing – review and editing (equal). **Masateru Senge:** Conceptualization (equal); writing – review and editing (equal).

## FUNDING INFORMATION

This study was supported by the Ministry of Education of Humanities and Social Science project (19YJAZH066) the Shanxi Scholarship Council of China (2020‐138), the key research base project of Humanities and Social Sciences in Shanxi Province (20200133); the Scientific and Technological Innovation Programs of Higher Education Institutions in Shanxi (2019L0781 and 2021L436), and the Shanxi Federation of Social Sciences (2020YY207) and the Shanxi graduate education teaching reform topic (2022YJJG245).

## CONFLICT OF INTEREST STATEMENT

The authors declare that there are no conflicts of interest regarding the publication of this paper.

## ETHICS STATEMENT

Not applicable.

## CONSENT TO PARTICIPATE

Not applicable.

## CONSENT FOR PUBLICATION

Not applicable.

## CODE AVAILABILITY

Not applicable.

## Data Availability

All data generated or analyzed during this study are included in this published article.
